# Higher physical activity level and perceived social support is associated with less psychological distress in people with anxiety

**DOI:** 10.7717/peerj.16000

**Published:** 2023-11-02

**Authors:** Damián Pereira-Payo, Ángel Denche-Zamorano, Noelia Mayordomo-Pinilla, Juan Manuel Franco-García, Antonio Castillo-Paredes, Miguel Angel Garcia-Gordillo, Jorge Rojo-Ramos, Sabina Barrios-Fernández

**Affiliations:** 1 Promoting a Healthy Society Research Group (PHeSO), Faculty of Sport Sciences, University of Extremadura, Cáceres, Extremadura, Spain; 2 Health, Economy, Motricity and Education (HEME) Research Group, Faculty of Sport Sciences, University of Extremadura, Cáceres, Extremadura, Spain; 3 Grupo AFySE, Investigación en Actividad Física y Salud Escolar, Escuela de Pedagogía en Educación Física, Facultad de Educación, Universidad de Las Americas, Santiago, Santiago, Chile; 4 Universidad Autónoma de Chile, Talca, Chile; 5 Physical Activity for Education, Performance and Health, Faculty of Sport Sciences, University of Extremadura, Cáceres, Extremadura, Spain; 6 Occupation, Participation, Sustainability and Quality of Life (Ability Research Group), Nursing and Occupational Therapy College, University of Extremadura, Cáceres, Extremadura, Spain

**Keywords:** Health survey, Psychology, Mental health, Stress, Successful coping, Self-steem

## Abstract

**Introduction:**

Anxiety is one of the most prevalent mental illnesses in first world societies, generating discomfort in the people who suffer from it, and high expenses and economic losses in the society. The physical activity (PA) performed, together with the perceived social support (PSS) by people with anxiety could be related to the psychological distress of people with anxiety.

**Objectives:**

To study the relationships between mental health and its dimensions, through Golberg’s General Health Questionnaire (GHQ12), and the level of PA (PAL) and the PSS in the Spanish adult population with anxiety.

**Hypothesis:**

A higher PAL, and a higher PSS, is related to a lower psychological distress in this population.

**Design and Methodology:**

This study included 1,661 adults with anxiety, residents in Spain. It was a cross-sectional study with data obtained from the Spanish National Health Survey. A Kolmogorov–Smirnov test was applied to examine the data distribution of the variables. The median and interquartile range were used to characterize the sample for continuous variables, and absolute and relative frequencies were used for categorical variables. The Mann–Whitney U test was used to examine the differences between sexes. Dependence between PAL and sex was studied using the chi-square statistic. A Krustal–Wallis test was used to evaluate the existence of differences in the baseline medians on the GHQ-12, according to PAL level. Finally, the correlations between mental health and its dimensions with PAL and the Duke-UNC-11 scores were analysed, obtaining Spearman’s rho and Pearson’s correlation coefficient.

**Results:**

Moderate inverse correlations were found between the GHQ-12 and: PAL (rho: −0.219); PSS (r: −0.347). PAL and PSS presented moderate inverse correlations with successful coping (rho: −0.206 and r: −0.325), self-esteem (rho: −0.222 and r: −0.333) and stress (rho: −0.158 and r: −0.288).

**Conclusions:**

Greater PAL and social support are associated with reduced psychological distress in people with anxiety.

## Introduction

The World Health Organization (WHO), in its pursue of universal health coverage, assigns mental health an essential role in its current concept of health (World Health Organization, 2019). As defined by the World Health Organization (WHO), mental health is a state of wellbeing where an individual is aware of their own abilities, is able to manage normal life stress, is productive and able to contribute to their community. From this definition, it can be inferred that mental health is a crucial aspect of overall health and goes beyond the mere absence of illness, and that it is closely linked to both physical health and behaviour (World Health Organization, 2004).

Unfortunately, there are factors that disrupt mental health and undermine it, causing disorders such as anxiety (Kroenke et al., 2007). Most common mental disorders can be classified in to two main diagnostic categories: depressive disorders and anxiety disorders (World Health Organization, 2017). Anxiety disorders are a classification of mental health conditions marked by persistent and excessive feelings of anxiety and fear (World Health Organization, 2017). Similarly to depression, symptoms can range from mild to severe, and the common duration of the symptomatology makes it more a chronic than an episodic disorder (World Health Organization, 2017). Anxiety disorders are characterized for experiencing a disproportionate fear and worry to actual threat that interferes sufferers normal functioning (Olthuis et al., 2016). These disorders translate into a variety of physical, cognitive, emotional, and behavioural symptoms that negatively affect the patient functioning, quality of life and wellbeing (Kandola et al., 2018; Olthuis et al., 2016; Simpson et al., 2010). Some of these symptoms are rapid breathing, tense muscles, irritability, difficulty concentrating, difficulty sleeping and hyperarousal (Olthuis et al., 2016). The specific symptoms a patient experiences depends on the type of anxiety disorder he/she suffers from (Olthuis et al., 2016). Anxiety subtypes include: post-traumatic stress disorder, generalised anxiety disorder, social phobia, specific phobias, selective mutism, separation anxiety disorder, panic disorder, and agoraphobia (American Psychiatric Association, 2013; Kandola et al., 2018; Kessler et al., 2009; World Health Organization, 1992).

Anxiety disorders have large prevalence globally, some estimations go from 3.8% to 25% depending on the country, and increasing to 70% of individuals with chronic conditions (Kandola et al., 2018). According to the Global Burden of Disease Study, anxiety disorders are the 6th largest contributor to disability worldwide, accounting for 26.8 million years lived with this condition and ranking second globally (Baxter et al., 2014; Kandola et al., 2018; Xiong et al., 2022). Its prevalence is estimated to be higher in the more developed countries than in developing countries (Essau et al., 2018; Kessler et al., 2015), and it is more common among females than in males (World Health Organization, 2017). In Spain, the prevalence rate of mental disorders is 286.7 cases per 1,000 inhabitants, affecting more women than men (Ministry of Health, Consumer Affairs and Social Welfare of Spain, 2022). Among these mental disorders, the most prevalent is anxiety, with 74.6 cases per 1,000 inhabitants (Ministry of Health, Consumer Affairs and Social Welfare of Spain, 2022). The economic costs generated by anxiety are among the highest of all mental disorders, along with depression, accounting for 2.2 percent of the gross domestic product (GDP) of Spain (Ruiz-Rodriguez et al., 2017).

Globally 7.3% of individuals live with at least one anxiety disorder (Thibaut, 2022). These kind of disorders have been shown to be associated with substantial impairment, that increases as the patient suffers from more than one anxiety disorder (Kroenke et al., 2007). The DSM-5 categorizes the following as anxiety disorders: Substance/Medication-Induced Anxiety Disorder, Selective Mutism, Social Anxiety Disorder, Separation Anxiety Disorder, Panic Disorder, Agoraphobia, Other Specified Anxiety Disorder, Unspecified Anxiety Disorder, Anxiety Disorder caused by a medical condition, Generalized Anxiety Disorder and Specific Phobia (Morrison, 2015). One of the strongest comorbidities of anxiety is depression; among those with major depressive disorder, 45.7% have developed at least one anxiety disorder, and 41.6% have had it concurrently with substance abuse disorder (Kalin, 2020; Kessler et al., 2015). Several chronic conditions such as cancer, chronic pain, irritable bowel syndrome, asthma, and cardiovascular diseases have been associated with anxiety (McDowell et al., 2019; Roy-Byrne et al., 2008). When sufferers of any of this conditions also have anxiety, it has been shown that a worsening in the recovery and in some cases premature mortality may occur (McDowell et al., 2019; Roy-Byrne et al., 2008). Additionally, those who have suffered from a virus related to the SARS family are more likely to have a comorbidity with anxiety (Mak et al., 2009). The effects of anxiety diminish the quality of life of those who suffer from it, and can lead to disabilities and impairments that result in more visits to the healthcare system (Kroenke et al., 2007; Roy-Byrne et al., 2008).

Research has shown multiples benefits for PA, one of them is the positive effect of PA on mental disorders (Denche-Zamorano et al., 2022a; Galán-Arroyo et al., 2022a; Galán-Arroyo et al., 2022b; Saxena et al., 2005; Schuch et al., 2020; Warburton, Nicol & Bredin, 2006) and on the symptoms and problems derived from them, such as stress, self-esteem and resilience (Maugeri et al., 2020). PA has been proposed as a strategy to treat anxiety, various studies have corroborated that PA is effective combating anxiety, and reducing its symptomatology in those who suffer from it (Maugeri et al., 2020; McDowell et al., 2019; Rebar et al., 2015). With the advent of COVID-19, measures such as social isolation and quarantine were applied, these rules reduce the number of social interactions, increasing loneliness, which is one of the factors that increase the symptoms of depression and anxiety (Benke et al., 2020; Palgi et al., 2020). Additionally, quarantine limited the ability of citizens to perform PA, reducing active hours and increasing sedentarism (Castañeda-Babarro et al., 2020), which constitutes another risk factor, since individuals who do not perform PA are up to 40% more likely to develop an anxiety disorder (Schuch et al., 2020).

PSS can be defined as the experience of being valued, respected and supported by the social groups to which one individual belongs (Roohafza et al., 2014; Shumaker & Brownell, 1984), several studies show that PSS is related to anxiety and its symptomatology, so that a high level of PSS would improve an individual’s mental state and reduce anxiety (Cheval et al., 2021; Procidano & Smith, 1997; Roohafza et al., 2014). PSS has been found to be more important for mental health than social support during the COVID-19 pandemic (Gülaçtı, 2010). PSS has been proposed as a significant predictor of wellbeing as it reflects an individual’s subjective assessment of their resources and is a protective factor for mental health (Nauffal & Sbeity, 2013; Patrick, Cottrell & Barnes, 2001). Research suggests that proper PSS can reduce anxiety and depression symptoms (Cheval et al., 2021; Grey et al., 2020; Stanton et al., 2020). Additionally, PAL has been shown to have a positive impact on PSS in both adolescents and older adults (Kang, Park & Wallace, 2018; Yusuf et al., 2021).

The aim of this study was to examine the relationships between Physical Activity Level (PAL) and perceived social support as measured by the Duke-UNC-11 Functional Social Support Questionnaire, with mental health and its dimensions, according to Golberg’s General Health Questionnaire (GHQ-12), in a Spanish adult population with anxiety. The initial hypothesis was that people with a higher PAL and PSS score would show lower levels of psychological distress as indicated by their GHQ-12 scores.

## Materials & Methods

### Design

The current cross-sectional study aims to find associations between PAL and PSS with mental health, and its dimensions defined by the GHQ-12 questionnaire, in the data reported by the Ministry of Health, Consumption and Well-being (MSCBS) in the National Health Survey of Spain 2017 (ENSE 2017) (Ministry of Health, Consumer Affairs and Social Welfare of Spain, 2017). MCBS alongside the National Institute of Statistic (INE) carry out the ENSE every 5 years, with the purpose of knowing the health status of the Spanish residents. Interviews were conducted by certificate interviewers, who informed the participants about the confidentiality of the data and encourage them to participate. The interviews were done in October of 2016 and October of 2017.

### Participants

The sample of the ENSE 2017 was selected based on a random three-phase sampling system (Ministry of Health, Consumer Affairs and Social Welfare of Spain, 2017): 23,089 residents in Spain, 10,595 males and 12,494 females, all over the age of 15, were selected. The sample calculation, data processing, communication and acceptance of participants, as well as all relevant information about these procedures were fully described in the methodology of the ENSE 2017 (Ministry of Health, Consumer Affairs and Social Welfare of Spain, 2017). For this research, the following selection criteria were applied to the sample that made up the ENSE 2017 in order to select the final sample: self-reported anxiety, being under 70 years of age and presenting data on the following variables of interest: the GHQ-12 (items Q.47.1–Q.47.12), physical activity performed (items Q.113–Q.117) and the Duke-UNC-11 questionnaire (items Q.131.1–Q.131.11).

Final sample was composed by a total of 1,611 Spanish residents (516 men y 1,145 women) with chronic anxiety aged from 15 to 69 years old. To create this final sample, a total of 21.478 personas were excluded: 5,312 due to their age, because participants older than 70 years old, were not asked about their PAL in the ENSE 2017; 16.088 which did not have chronic anxiety (answered “No” to item 25.21.a), 24 people who did not have all data in the GHQ-12 (at least one of the following items were not answered: p.47.1–p.47.12), and four people whose PAL data was not complete (one or more of the following items were nor answered: p.113–p.117). Fifty-nine participants were not considered in the analysis of the Duke-UNC-11 results, because they have not answered every item in this questionnaire (p131.1–p.131.11).

### Variables

**Gender**: Male or female.

**Age**: In years.

**Mental health**: It was derived from the GHQ-12 questionnaire in its Spanish version (Goldberg & Williams, 1996), which is included in the ENSE 2017. It has 12 ítems, with four possible answers, every item can be scored from 0 to 3. In the GHQ-12, mental health can have overall scores from 0 to 36, which are obtained after adding up the score of every single item. A score of 0 represents the best mental health state and a score of 36, is the worst mental health state possible. In the ENSE 2017, GHQ-12 corresponds to the items p.47.1–p.47.12. This questionnaire is used to identify psychological distress and possible psychiatric disorders. Various studies have shown the reliability and validity of this instrument in the panish population, with a high internal consistency (*α* = 0,86) (Goldberg & Williams, 1996; Muñoz-Bermejo et al., 2020; Rocha, 2011; Sánchez, Pilar & Dresch, 2008).

Alongside mental health, the GHQ-12 allows us to evaluate three other dimensions, successful coping, self-esteem and stress (Muñoz-Bermejo et al., 2020; Sánchez, Pilar & Dresch, 2008).

**Successful coping (FI).** The variable was calculated by adding up the responses to items: P.47.1, P.47.3, P.47.4, P.47.7, P.47.8 and P.47.12, which are the questions 1, 3, 4, 7, 8 and 12 of the GHQ-12. Every item has four possible answers, and scores range from 0 to 3. Overall scores of this dimension go from 0 to 18, with 0 representing the best coping and 18 representing the worst coping. The factor has a validity of 0.82 and a *p*-value of 0.001 (Muñoz-Bermejo et al., 2020).

**Self-esteem (FII).** This variable was created by summing the answers to items P.47.6, P.47.9, P.47.10, and P.47.11 (Ministry of Health, Consumer Affairs and Social Welfare of Spain, 2017), which are the questions 6, 9, 10 and 11 of the GHQ-12. Every item has four possible options of response, and it scores range from 0 to 3. The overall responses score can range from 0 to 12, with 0 representing the highest level of self-esteem and 12 the lowest. The factor has an external validity of 0.70 and a *p*-value of 0.001 (Muñoz-Bermejo et al., 2020).

**Stress (FIII).** This variable was constructed with the sum of the answers to the items: P.47.2, P.47.5 y P.47.9, which correspond to the questions 2, 5 and 9 of the GHQ-12. These items have four options of response and are scored 0 to 3. Overall scores of the dimension stress can take values from 0 to 9, where 0 is less stress possible, and 9, the highest levels of stress. The validity of this factor is 0.75 with a *p*-value: 0.001 (Muñoz-Bermejo et al., 2020).

**Perceived social support (PSS):** This variable was formed summing the answers to the items 130.1–130.11 (Ministry of Health, Consumer Affairs and Social Welfare of Spain, 2017) from the ENSE 2017. These items correspond to the Duke-UNC-11 Functional Social Support Questionnaire, which evaluates participants perceived social support. It presents 11 items, with five possible answers each, that can take values from 0 (“Much less than I would like”) to 5 (“As much as I would like”). Thus, perceived social support is built summing all the answer, it can take values among 11 (which is the lower level of perceived social support) and 55 (which is the highest level of perceived social support). Scores under 32 show low perceived social support in Spanish population (Muñoz-Bermejo et al., 2020). This questionnaire has good internal consistency in this population (*α* = 0,90) (Broadhead et al., 1988; Muñoz-Bermejo et al., 2020).

**Physical Activity Index (PAI):** It was constructed from the items p.113–p.116, which are included in the ENSE 2017, and belong to the International Physical Activity Questionnaire (IPAQ) in its Spanish version (Craig et al., 2003). The respondents were asked about for how long and with which frequency they perform moderate and intense physical activity during a week. The PAI used, was an adaptation of the Physical Activity Index (Nes et al., 2011), it has the following formula: PAI = (Frequency of intense physical activity factor * Duration of intense physical activity factor) + (Factor for moderate physical activity intensity * Factor of moderate physical activity frequency * Factor of duration of moderate physical activity factor) and can take values between 0 and 67.5 (Denche-Zamorano et al., 2022b), with 0 meaning performing the less PA and 67.5 meaning performing the most PA.

**Physical Activity Levels (PAL):** There were stablished four levels of physical activity, taking into account the scores obtained in the PAI, and the answers to the item P.117 (*Now think about the time you spent walking in the last 7 days,* with the following possible answers: “*Any day more than 10 min at a time*”, or 1 to 7 days): Inactives (PAI = 0; Participants that reported not walking any day of the week for more than 10 min), Walkers (PAI = 0; Respondents that declared walking during 10 min or more at a time, at least one day of the week,), Actives (PAI = between 1 and 30) and Very actives (PAI over 30) (Denche-Zamorano et al., 2022b).

### Statistical analysis

Statistical procedures were performed with IBM SPSS Statistics software version 25, using a level of significance under 0.05.

The distributions followed by the data of the study variables were analyzed with the Kolgomorov-Smirnov test. The sample was characterized using the median and interquartile range (IQR) for the continuous variables (Age, PAI, Sucessfull-coping, Self-steem, Stress, Perceived social support and Mental health), analyzing the possible differences between sexes, using the Mann–Whitney U test, and the absolute and relative frequencies for the categorical variable (PAL), analyzing its dependence on sex, using the chi-square statistic. The mental health scores, as well as their dimensions, were presented by median and IQR, for each PAL group, analyzing possible differences in their baseline, both in the general population and by sex, using the Kruskal-Wallis test. A study of the correlations between PAL, and PSS, with GHQ-12 scores and items was carried out, using the correlation coefficients of Spearman and Pearson, and the correction of Bonferroni which was also applied as required. To predict the scores on: stress, self-esteem, successful coping and mental health (according to GHQ-12); and using sex, age, PAL, BMI and PSS as independent variables, linear regressions were used. The authors considered two-sided *p*-values ≤ 0.05 as statistically significant. All analyses were performed using IBM SPSS Statistics v.25 statistical software (SPSS Inc., Chicago, IL, USA).

## Results

The Kolgomorov-Smirnov test showed that there was not sufficient evidence to assume that the variables of study followed a normal distribution (*p* < 0.001).

Statistically significant differences were found in the PA of men and women (*p* = 0.006), according to PAI. Despite having identical medians, the mean was higher in men than in women (8.5 *vs.* 6.0). In this line, the association between PAL and sex was also found (*p* = 0.004). No significant differences were found between sexes in the variables derived from the GHQ-12: mental health (14 *vs* 14. *p* = 0.499), successful coping (7 *vs* 7. *p* = 0.098) and stress (4 *vs* 4. *p* = 0.744). Regarding PSS, the median was slightly higher in women (47 *vs* 46. *p* = 0.110) but significant differences neither existed, (Table 1).

**Table 1:** Descriptive analysis: age, PAI, dimensions-subscales GHQ-12, Duke-UNC-11 and PAL; Spanish adult with anxiety, ENSE 2017.

Variables	Total *n* = 1661	Men *n* = 516	Women *n* = 1145	*p*
Age (Years)				0.004a
Median (IQR)	53 (18)	51 (16)	53 (18)	
Mean (SD)	50.5 (12.3)	49.4 (11.6)	51.0 (12.5)	
PAI				0.006a
Median (IQR)	0 (0)	0 (15)	0 (0)	
Mean (SD)	6.8 (13.6)	8.5 (15.6)	6.0 (12.6)	
Mental health				0.499a
Median (IQR)	14 (9)	14 (10)	14 (9)	
Mean (SD)	15.6 (6.0)	15.8 (7.1)	15.5 (6.8)	
Successful coping				0.098a
Median (IQR)	7 (3)	7 (4)	7 (3)	
Mean (SD)	7.9 (2.9)	8.1 (3.0)	7.8 (2.9)	
Self-steem				0.744a
Median (IQR)	4 (5)	4 (5)	4 (5)	
Mean (SD)	4.5 (3.1)	4.6 (3.2)	4.5 (3.1)	
Stress				0.935a
Median (IQR)	5 (3)	4 (3)	5 (3)	
Mean (SD)	4.6 (2.3)	4.6 (2.4)	4.7 (2.3)	
Perceived social support	Total *n* = 1608	Men *n* = 494	Women *n* = 1114	0.110a
Median (IQR)	47 (13)	46 (14)	47 (11)	
Mean (SD)	45.0 (9.5)	44.3 (10.1)	45.3 (9.1)	
PAL				<0.004b
Inactives	364 (21.9%)	114 (22.1%)	250 (21.8%)	
Walkers	858 (51.7%)	246 (47.7%)	612 (53.4%)	
Actives	342 (20.6%)	111 (21.5%)	231 (20.2%)	
Very actives	97 (5.8%)	45 (8.7%)	52 (4.5%)	

**Notes.**

SD Standard deviation IQR Interquartile range % porcentage n participants PAL Physical Activity Level

a *p*-value from U-Mann–Whitney test.

b *p*-value from chi square test.

Inactive (PAI = 0; Subjects that do not walk any day of the week during 10 min at a time or more). Walkers (PAI = 0; Subjects that walk at least one day of the week during 10 min at a time or more). Actives (PAI = 1 to 30); Very actives (PAI = over 30); PAI (Physical Activity Index: Scores among 0 and 67.5); GHQ-12 (Goldberg’s General Health Questionnaire. Scores range from 0 to 36. Being 0, best mental health and 36, worst mental health); Stress (Scores range from 0 to 9. Being 0 no stress, and 9 very stressed); Self-esteem (Scores range from 0 to 9. Being 0 the best self-esteem, and 9 the worst self-esteem); Successful Coping (Scores go from 0 to 18. 0 is the best coping and 18 is the worst coping).

The Inactive group presented the highest scores, both in mental health and in its three dimensions, while the lowest score was in the Very active group, this was reported among both sexes and in the general population. For mental health scores, there was a 5-point difference in the medians between the Inactive and Very Active groups (17 *vs.* 12). The Krustal–Wallis test showed differences in the medians obtained in the GHQ-12 between the different PAL groups (*p* < 0.001), both in mental health and in its three dimensions (Table 2) and in the general population and by sex.

**Table 2:** Associations between PAL and dimensions-subscales of the GHQ-12 in Spanish adult with anxiety, ENSE 2017.

Variables	Total *n* = 1661			Men *n* = 516			Women *n* = 1145		
**Mental health**									
PAL	m (sd)	mdn (IQR)	*p*	m (sd)	mdn (IQR)	*p*	m (sd)	mdn (IQR)	*p*
Inactives	18.2 (7.7)	17 (12)	<0.001	18.8 (8.2)	17 (12)	<0.001	17.9 (7.4)	17 (11)	<0.001
Walkers	15.6 (6.7)	14 (9)		15.7 (6.7)	14 (9)		15.5 (6.7)	14 (8)	
Actives	13.8 (6.1)	12 (8)		14.4 (6.5)	13 (10)		13.6 (5.9)	12 (8)	
Very actives	12.6 (5.0)	12 (7)		12.5 (5.1)	12 (7)		12.8 (4.9)	12 (7)	
**Successful coping**									
PAL	m (sd)	mdn (IQR)	*p*	m (sd)	mdn (IQR)	*p*	m (sd)	mdn (IQR)	*p*
Inactives	9.0 (3.4)	9 (6)	<0.001	9.3 (3.7)	8 (6)	<0.001	8.8 (3.3)	8 (5)	<0.001
Walkers	7.9 (2.8)	7 (4)		8.1 (2.8)	7 (4)		7.8 (2.8)	7 (3)	
Actives	7.1 (2.4)	7 (3)		7.5 (2.4)	7 (3)		7.0 (2.3)	6 (2)	
Very actives	6.5 (1.9)	6 (2)		6.5 (2.2)	6 (2)		6.6 (1.7)	6 (1)	
**Self-steem**									
PAL	m (sd)	mdn (IQR)	*p*	m (sd)	mdn (IQR)	*p*	m (sd)	mdn (IQR)	*p*
Inactives	5.7 (3.3)	5 (5)	<0.001	5.9 (3.4)	6 (6)	<0.001	5.6 (3.3)	5 (5)	<0.001
Walkers	4.5 (3.0)	4 (4)		4.5 (3.0)	4 (5)		4.5 (3.0)	4 (4)	
Actives	3.8 (3.0)	3 (5)		3.8 (3.2)	3 (5)		3.7 (2.9)	3 (5)	
Very actives	3.2 (2.3)	3 (4)		3.2 (2.3)	3 (3)		3.2 (2.4)	3 (4)	
**Stress**									
PAL	m (sd)	mdn (IQR)	*p*	m (sd)	mdn (IQR)	*p*	m (sd)	mdn (IQR)	*p*
Inactives	5.2 (2.3)	5 (4)	<0.001	5.3 (2.4)	6 (4)	0.001	5.2 (2.3)	5 (4)	<0.001
Walkers	4.6 (2.3)	5 (3)		4.6 (2.4)	5 (3)		4.6 (2.2)	5 (3)	
Actives	4.2 (2.3)	4 (4)		4.3 (2.4)	4 (4)		4.2 (2.3)	4 (4)	
Very actives	4.0 (2.3)	4 (4)		3.9 (2.2)	3 (4)		4.2 (2.3)	4 (3)	

**Notes.**

SD Standard deviation m mean IQR Interquartile range mdn median p *p*-value from Kruskal-Wallis test PAL Physical Activity Level GHQ-12 Goldberg’s General Health Questionnaire

Inactive (PAI = 0; Subjects that do not walk any day of the week during 10 min at a time or more). Walkers (PAI = 0; Subjects that walk at least one day of the week during 10 min at a time or more). Actives (PAI = 1 to 30); Very actives (PAI = over 30); PAI (Physical Activity Index: Scores among 0 and 67.5). Scores range from 0 to 36. Being 0, best mental health and 36, worst mental health); Stress (Scores range from 0 to 9. Being 0 no stress, and 9 very stressed); Self-esteem (Scores range from 0 to 9. Being 0 the best self-esteem, and 9 the worst self-esteem); Successful Coping (Scores go from 0 to 18. 0 is the best coping and 18 is the worst coping).

The correlations among PAL and the variables derived from the GHQ-12 are shown in Table 3. Weak inverse correlations were found between PAL and: stress (rho: −0.158. *p* < 0.001), self-confidence (rho: −0.222. *p* < 0.001), successful coping (rho: −0.216. *p* < 0.001) and mental health (rho: −0.219. *p* < 0.001) (Mondragón & Alejandra, 2014). Small inverse correlations were also found among PAL and GHQ-12 items (Table 3).

**Table 3:** Correlation between PAL and Goldberg General Health Questionnaire (GHQ-12) in Spanish adults with anxiety.

**Target variable**	**Rho**	***p***
Mental health	−0.219	<0.001
Successful coping	−0.206	<0.001
Self-steem	−0.222	<0.001
Stress	−0.158	<0.001
1. Have you been able to concentrate well on what you were doing?	−0.144	<0.001
2. Have your worries caused you to lose sleep?	−0.098	0.002
3. Did you feel that you were playing a useful role in life?	−0.144	<0.001
4. Did you feel able to make decisions?	−0.180	<0.001
5. Have you felt constantly overwhelmed and under stress?	−0.139	<0.001
6. Have you had the feeling that you cannot overcome your difficulties?	−0.170	<0.001
7. Have you been able to enjoy your normal daily activities?	−0.193	<0.001
8. Have you been able to cope adequately with your problems?	−0.205	<0.001
9. Have you felt unhappy or depressed?	−0.168	<0.001
10. Have you lost confidence in yourself?	−0.205	<0.001
11. Have you thought of yourself as a worthless person?	−0.219	<0.001
12. Do you feel reasonably happy considering all the circumstances?	−0.172	<0.001

**Notes.**

p *p*-value PAL Physical Activity Level

Rho (Spearman’s correlation coefficients with the Bonferroni correction factor having (*p* = 0.003); Inactive (PAI = 0; Subjects that do not walk any day of the week during 10 min at a time or more). Walkers (PAI = 0; Subjects that walk at least one day of the week during 10 min at a time or more). Actives (PAI = 1 to 30); Very actives (PAI = over 30); PAI (Physical Activity Index: Scores among 0 and 67.5); GHQ-12 (Goldberg’s General Health Questionnaire. Scores range from 0 to 36. Being 0, best mental health and 36, worst mental health); Stress (Scores range from 0 to 9. Being 0 no stress, and 9 very stressed); Self-esteem (Scores range from 0 to 9. Being 0 the best self-esteem, and 9 the worst self-esteem); Successful Coping (Scores go from 0 to 18. 0 is the best coping and 18 is the worst coping).

Finally, Table 4 shows the correlations among PSS and the variables derived from the GHQ-12. Moderate inverse correlations were found between PSS and: stress (r: −0.288. *p* < 0.001), self-confidence (r: −0.333. *p* < 0.001), successful coping (r: −0.325. *p* < 0.001), and mental health (r: −0.347. *p* < 0.001) (Mondragón & Alejandra, 2014). In addition, weak correlations were found between PSS and GHQ-12 items (Table 4) (Mondragón & Alejandra, 2014).

**Table 4:** Correlation between perceived social support (Duke-UNC-11) and the Goldberg General Health Questionnaire (GHQ-12) in Spanish adults with anxiety.

**Target variable**	**Correlations**	***p***
Mental health	−0.347^*^	<0.001
Successful coping	−0.325^*^	<0.001
Self-steem	−0.333^*^	<0.001
Stress	−0.288^*^	<0.001
1. Have you been able to concentrate well on what you were doing?	−0.185^**^	<0.001
2. Have your worries caused you to lose sleep?	−0.195^**^	<0.001
3. Did you feel that you were playing a useful role in life?	−0.189^**^	<0.001
4. Did you feel able to make decisions?	−0.182^**^	<0.001
5. Have you felt constantly overwhelmed and under stress?	−0.237^**^	<0.001
6. Have you had the feeling that you cannot overcome your difficulties?	−0.233^**^	<0.001
7. Have you been able to enjoy your normal daily activities?	−0.248^**^	<0.001
8. Have you been able to cope adequately with your problems?	−0.239^**^	<0.001
9. Have you felt unhappy or depressed?	−0.275^**^	<0.001
10. Have you lost confidence in yourself?	−0.254^**^	<0.001
11. Have you thought of yourself as a worthless person?	−0.234^**^	<0.001
12. Do you feel reasonably happy considering all the circumstances?	−0.264^**^	<0.001

**Notes.**

* Pearson’s correlation coefficients with the Bonferroni correction factor having *p* = 0.003.

** Spearman’s correlation coefficients with the Bonferroni correction factor having *p* = 0.003.

p *p*-value GHQ-12 Goldberg’s General Health Questionnaire

Scores range from 0 to 36. Being 0, best mental health and 36, worst mental health); Stress (Scores range from 0 to 9. Being 0 no stress, and 9 very stressed); Self-esteem (Scores range from 0 to 9. Being 0 the best self-esteem, and 9 the worst self-esteem); Successful Coping (Scores go from 0 to 18. 0 is the best coping and 18 is the worst coping). Duke-UNC-11 (Duke-UNC-11 Functional Social Support Questionnaire. Scores go from 11 to 55 points).

Table 5 show the linear regression models to predict the GHQ-12 (mental health and its factors) scores, based on the variables: Sex, Age, BMI, PSS and PAL.

## Discussion

The main purpose of this study was to explore the relationship among PAL, PSS and mental health in Spanish adults with chronic anxiety, living in Spain. The core result was that significant connections were discovered between PAL, PSS, and GHQ-12 scores. Psychological distress, as defined by GHQ-12, was found to be reduced in people with higher PAL, the same occurred in all dimensions of mental health assessed with this questionnaire, where higher physical activity was associated with greater mental health.

**Table 5:** Linear regression analysis for mental health and its factor, with: sex, age, IMC, PSS and PAL; like independent variables.

Mental health				
	*β*	t	*p*	R^2^
PAL	−1.633	−8.172	<0.001	15.5%
PSS	−0.238	−1.603	<0.001	
Constant	29.696	37.715	<0.001	
Successful coping				
	*β*	t	*p*	R^2^
PAL	−0.706	−8.283	<0.001	14.3%
PSS	−0.095	−12.667	<0.001	
Constant	13.602	37.279	<0.001	
Self-steem				
	*β*	t	*p*	R^2^
PAL	−0.721	−7.873	<0.001	14.2%
PSS	−0.103	−12.844	<0.001	
Constant	10.646	27.134	<0.001	
Stress				
	*β*	t	*p*	R^2^
PAL	−0.369	−5.316	<0.001	9.8%
PSS	−0.068	−11.134	<0.001	
Constant	8.448	28.429	<0.001	

**Notes.**

PAL Physical Activity Level PSS Perceived Social Support B Understandarized beta t *t*-value p *p*-value R^2^ Nagelkerke’s R Square

Males presented significantly higher PA than females reflected in the PAI mean (8.5 *vs* 6.0) (p: 0.006). However, the medians for both sexes and the general population were zero. The distribution by groups in the PAL was similar in the general population and by sexes, with the Walkers group (general = 858 (51.7%); men = 246 (47.7%); women = 612 (53.4%)) being the most numerous and the Very Actives (general = 97 (5.8%); men = 45 (8.7%); women = 52 (4.5%)) being the least populated, likewise the percentage of participants in the groups Inactives (general = 364 (21.9%); men = 114 (22.1%); women = 250 (21.8%)) and Actives (general = 342 (20.6%); men = 111 (21.5%); women = 231 (20.2%)) was similar. On the other hand, associations were found between PAL and sex (p: 0.004). A larger proportion of males than females was found in the very active level (8.7% *vs* 4.5%). In contrast, the proportion of women in the walkers group was higher than the proportion of men (53.4% *vs* 47.7%). This suggest that more males tend to get involved in physical activity of higher intensity and with greater frequency than females, but when looking at the individuals who only walk, it would be the other way around. Some research supports these findings, reporting that men are more active and prefer activities of higher intensity, while women perform more moderate PA such as walking (Abel, Graf & Niemann, 2001; Hernández Álvarez et al., 2010). Anxiety sensitivity has been proposed as a potential mediator in gender differences in PA, and one of the causes of women having lower levels of PA (DeWolfe et al., 2019). On the other hand, several studies do not find significant differences in the type of physical activity performed by gender, and report that the most frequent activity in both sexes is walking (Azevedo et al., 2007; Ceballos Gurrola, Bermúdez & Rodríguez, 2012; Lee, 2005).

The median PSS was slightly higher in women than in men (47 *vs* 46), although no statistically significant differences were found between sexes. Supporting these results, other investigations did not find any differences in PSS between sexes (Barnett et al., 2021). Moderate opposite correlations were found among PSS and mental health (r: −0.347. *p* < 0.001) and its three dimensions successful coping (r: −0.325. *p* < 0.001), self-confidence (r: −0.333. *p* < 0.001) and stress (r: −0.288. *p* < 0.001). Other researches have reported similar associations between PSS and mental health (Adamczyk & Segrin, 2015; Singh, Verma & Lata, 2022). Moderate inverse correlations have been reported between PSS and anxiety and depression (Guo, Tan & Zhu, 2022), and also small direct correlations among PSS and mental health (Dong et al., 2022). In this line other investigations found that mental health (evaluated by GHQ-12), successful coping, self-confidence and stress were inversely correlated to PSS (Denche-Zamorano et al., 2022d; Denche-Zamorano et al., 2022e; Franco-García et al., 2023) in various populations. This is in line with the results of the present study and could suggest that higher PSS is associated with greater mental well-being (Adamczyk & Segrin, 2015; Dong et al., 2022; Guo, Tan & Zhu, 2022; Singh, Verma & Lata, 2022). Furthermore, small inverse correlations were found among PSS and all the GHQ-12 items, which may indicate that greater PSS is associated with lower scores in the mental health variables derived from the GHQ-12, Similar results were found in a population of people with depression (Denche-Zamorano et al., 2022d), asthma (Denche-Zamorano et al., 2022e) and people with cancerous tumours (Franco-García et al., 2023) where PSS had significant inverse correlations with the GHQ-12 items. While correlations among PSS and GHQ-12 items, and its dimensions (mental health, successful copping, self-esteem and stress) has been found, and it suggests that greater PSS is associated with a better state of the mentioned dimensions, cause–effect relationships cannot be stablished at the light of the present results.

Regarding mental health and the three dimensions that integrated it (successful coping, self-steem and stress) no significant differences between sexes existed, a median of 14 points was found for the general population and for both sexes. But among PA groups, differences in mental health and its dimensions were reported. In fact, the Inactives group presented the highest scores in all four variables, while the Very actives group had the lowest scores among PA groups. This could indicate that the inactive Spanish adults with anxiety may have a poorer mental health, self-steem, successful coping ability and the highest stress levels, and also that the Very actives may have a better state regarding mental health and the three dimensions that formed it. Moreover, small inverse correlations between PAL and mental health (rho: −0.347, *p* < 0.001) and its three dimensions, successful coping (rho: −0.325, *p* < 0.001), self-steem (rho: −0.333, *p* < 0.001) and stress (rho: −0.288, *p* < 0.001) were found. This could indicate that having greater PAL is associated with lower scores in all mental health variables studied, which would mean greater mental well-being. Additionally, a linear regression model showed that PAL combined with PSS predict mental health in a 15.5% (*β* = −1.633; *t* = −8.172; p = <0.001), successful coping in a 14.3% (*β* = −0.706; t = −8.283; *p* = <0.001), self-steem in a 14.2% (*β* = −0.721; t = −7.873; *p* = <0.001) and stress in a 9.8% (*β* = −0.369; t = −5.316; *p* = <0.001). All this suggests that Spanish adults with anxiety that perform larger PA could be more prone to have good mental health, self-steem, successful coping ability and less stress. In research PA and exercise have been commonly associated with greater mental wellbeing (Fox, 1999); thus, PA activity has been shown to protect and prevent against anxiety symptoms and anxiety (Fox, 1999; McDowell et al., 2019), even regardless of demographic factors (Schuch et al., 2019). In line with the present results, a dose–response relationship between PA and psychological distress, where greater physical activity means better psychological condition seems to exist, but there is not an absolute consensus about it (McDowell et al., 2019). Suggestions for the best amount and highest limit of physical activity to decrease anxiety symptoms have been proposed for the general population (Kim et al., 2020), and both resistance and aerobic exercise have been shown to improve anxiety and psychological distress (LeBouthillier & Asmundson, 2017). In line with the present findings, PA has been also reported to be inversely correlated with successful coping and self-esteem in adults with asthma (Denche-Zamorano et al., 2022e), the same was found for these two variables and also for stress in adults with depression (Denche-Zamorano et al., 2022d), in adults with cancerous tumours (Franco-García et al., 2023) and in a population of informal caregivers (Denche-Zamorano et al., 2022c). Successful coping ability (Craven et al., 2022; Dahlstrand et al., 2021; Denche-Zamorano et al., 2022d; Denche-Zamorano et al., 2022e), stress (Denche-Zamorano et al., 2022c; Denche-Zamorano et al., 2022d) and self-steem (Denche-Zamorano et al., 2022c; Denche-Zamorano et al., 2022d) seem to be improved by performing PA, which supports the available research that suggests the potential of PA on the prevention of depression, anxiety and other mental disorders (Harvey et al., 2010; Herring, Lindheimer & O’Connor, 2014; Ji et al., 2022; Kandola et al., 2018; Lautenschlager et al., 2004; Schuch et al., 2019; Stubbs et al., 2017; Wijndaele et al., 2007). Despite the correlations found among PAL and GHQ-12 items and dimensions, and the available research supporting that performing greater PA is associated with a better mental state, cause–effect relationships cannot be stablished between performing larger PA and a better state in the dimensions evaluated by GHQ-12, including mental health.

## Limitations

The current study presented the limitations inherent to a cross-sectional research design. The main limitation of the current research was that causal relationships could not be set, so it would be convenient to carry out other types of study designs that could establish both optimal doses and causal relationships. No objective measures were available to quantify either the amount or the intensity of PA performed by the participants. It would be advisable to include PA measurement devices in the respondents of future ENSE. Some sociodemographic biases that could influence the results were also not taken into account, such as: level of education, socioeconomic level and rural or urban living area.

## Practical applications

This study analyses the association among mental health, PAL and PSS in Spanish adults with chronic anxiety that live in Spain, it may create a reference system for future studies regarding mental health in this population. PAL and PSS are proposed as protective factors against mental health difficulties. Reducing psychological distress of people with anxiety may be key in order to decrease the health care costs derived from this disorder, especially given its prevalence in the Spanish adult population (Villagrasa et al., 2019). Several studies support the potential effect of PA (Harvey et al., 2010:20; Herring, Lindheimer & O’Connor, 2014; Kandola et al., 2018; Lautenschlager et al., 2004; McDowell et al., 2019; Schuch et al., 2019; Stubbs et al., 2017) and good levels of PSS (Adamczyk & Segrin, 2015; Dong et al., 2022; Guo, Tan & Zhu, 2022; Singh, Verma & Lata, 2022), improving mental health, and even the positive effect of greater PAL on PSS (Kang, Park & Wallace, 2018; Yusuf et al., 2021), but cause–effect associations cannot be established due to the design of our study. Thus, it seems that disseminating the importance of good social support networks for people with anxiety and promoting the relevance of having a physically active lifestyle in this population is a relevant action that should be taken from the institutions.

## Conclusions

According to the presented results, it can be concluded that an association among mental health and its three dimensions (Sucessfull-coping, Self-steem and Stress) and PAL and PSS in the Spanish adult population with anxiety exists. Higher PAL and PSS individuals present lower psychological distress, evaluated by the GHQ-12. Thus, research to come should consider addressing the effects of physical activity centered interventions as a lifestyle strategy and a complementary treatment to prevent and improve psychological distress in adults with anxiety.

## Additional Information and Declarations

### Data Availability

The following information was supplied regarding data availability: The data is available at Encuesta Nacional de Salud de España 2017: https://www.mscbs.gob.es/estadEstudios/estadisticas/encuestaNacional/encuesta2017.htm .
